# The relationship between energy expenditure and physical functions in patients hospitalised for stroke

**DOI:** 10.1038/s41598-021-01135-3

**Published:** 2021-11-04

**Authors:** Kota Hobo, Hideaki Kurita, Kimito Momose

**Affiliations:** 1 Department of Rehabilitation, Setagaya Rehabilitation Hospital, 6-37-1 Matsubara, Setagaya, Tokyo, 156-0043 Japan; 2 Department of Physical Therapy, School of Health Sciences, Tokyo University of Technology, Tokyo, Japan; 3 Department of Physical Therapy, School of Health Sciences, Shinshu University, Matsumoto, Japan

**Keywords:** Health occupations, Medical research

## Abstract

We assessed the relationship between energy expenditure (EE) and Functional Independence Measure motor items (FIM-M) score, Berg Balance Scale (BBS) score, and comfortable walking speed (CWS) in patients hospitalised for stroke. The total EE per day (TEE), EE during rehabilitation (REE), and EE during activities other than rehabilitation (OEE) were measured using a single-axis acceleration sensor in 36 patients hospitalised for the first stroke episode. In addition, the relationships between each type of EE and FIM-M, BBS, and CWS were investigated. In these patients (mean age 66.2 ± 10.6 years), the median values of TEE, REE, and OEE were 41.8 kcal, 18.5 kcal, and 16.6 kcal, respectively. Correlations were observed between each EE type and all physical function indices. Following the stratification of patients into two groups (high and low) based on the level of physical function, a significant correlation between EE type and physical function was observed only in the low BBS group. EE was correlated with overall physical function indices, but the trend differed depending on physical ability. When patients were stratified based on ability, there were several groups with no significant correlation. Therefore, several patients were unable to achieve an appropriate EE for their level of physical function.

## Introduction

People with low daily physical activity experience deterioration of physical function^1^, whereas those with high physical activity have lower mortality rates and lower incidence of ischemic heart disease and hypertension^2–4^. In addition, physical activity centred on walking is known to reduce mortality rates in older adults^5^, emphasising the importance of physical activity in an upright position. Physical activity is any movement of the body’s skeletal muscles that causes energy expenditure (EE), including exercise, housework, and work^6^. The Physical Activity Standard for Health Promotion 2013^7^ established by the Ministry of Health, Labour, and Welfare recommends that persons older than 65 should perform 40 min of physical activity every day. A study in which the EE of older adults was calculated using a questionnaire^8^ reported that physical activity of 1,000 kcal (142 kcal/day) or more for at least 1 week improved physical function.

Prolonged confinement to the bed and sitting has been related to decreased physical function in stroke patients^9,10^ Hence, stroke patients must engage in daily physical activity to maintain and improve function.

A study on stroke patient activity found that more than half of stroke patients in the community setting had low physical activity, below the 142 kcal/day recommended for older adults^11^. Furthermore, it has been reported that hospitalised stroke patients have spent less time being physically active^12–14^. Thus, although stroke often causes physical ability to deteriorate, these patients do not engage in an appropriate amount of physical activity.

The type of exercise also contributes to increasing physical function. A previous study of stroke patients within 6 months after onset^15^ found that physical activity time in an upright position, such as standing or walking, was positively correlated with the Berg Balance Scale (BBS) and Barthel Index (BI). In addition, compared with conventional therapy, increased physical activity time in an upright position during rehabilitation improved gait function^16^. Further, walking speed, Functional Independence Measure motor items (FIM-M), BI, and other indices have been reported to improve with increased rehabilitation time and activity in an upright position^17,18^.

What is the best way to quantify physical activity? A previous review assessing the amount of physical activity in stroke patients^19^ analysed studies that used equipment (accelerometer and pedometer) to measure physical activity. Physical activity in these studies comprised activity time, activity intensity, and the number of activities over a 3-day period, all of which are simple and reliable to measure in a clinical setting^19^. An accelerometer is an objective, reliable, and valid tool to measure activity in stroke patients^11^, and a significant correlation between metabolic equivalents obtained from uniaxial accelerometers and calorimetry in patients with brain injury has been shown^20^. Physical activity is often measured using activity time and not EE. However, physical activity refers to all activities that lead to EE^6^. Importantly, activity time does not reflect the intensity of the activity. For example, EE changes when walking speed is adjusted even if the walking time remains the same^21,22^. Therefore, EE better reflects physical activity than activity time.

Stroke patients should increase their overall physical activity. Of the total EE (TEE) per day in hospitalised stroke patients, EE during rehabilitation (REE) is critical, but it is also important to increase EE at times other than during rehabilitation (OEE); however, no studies have specifically examined REE and OEE. Therefore, the novelty of this study is that it is the first to measure the TEE, REE, and OEE in subacute stroke patients and assess their relationship with physical function indices (FIM-M, BBS, and comfortable walking speed [CWS]). Therapists should attempt to increase REE, since it has been obvious that increased rehabilitation in an upright position improves physical function^16–18^. However, therapists cannot increase OEE alone. Therefore, we hypothesised that there is a correlation between REE, but not OEE, and physical function. Hence, this study aimed to understand the current status of REE and OEE in hospitalised stroke patients and investigate the relationship with physical function. The clinical implication of this study was to demonstrate the importance of rehabilitation programme planning using quantity as an index rather than the quality of physical activity, considering the intensity of the activity.

## Methods

### Target population

A cross-sectional study was conducted on first-stroke patients who were hospitalised in a convalescent rehabilitation ward. For the CWS measurement, patients were required to walk 16 m or more without physical assistance, regardless of whether they had walking aids or orthosis; therefore, each therapist in charge evaluated and included such patients. Exclusion criteria included neurological disorders other than stroke that significantly limited movement, cardiovascular disorders, orthopaedic disorders, cognitive dysfunction and higher brain dysfunction that markedly decreased motivation (apathy scale 16 points or more)^23^, and sleep disorders (see Supplementary Fig. S1 for a flowchart of the study design).

### Activity measurement

Vertical acceleration was measured using a single-axis accelerometer (Lifecorder, Suzuken Co. Ltd, Nagoya, Japan), measuring 62 × 46 × 26 mm and weighing 40 g. The sampling frequency was 32 Hz, and the range of acceleration was 0.06–1.94 g (1.00 g is the acceleration of free fall: 9.807 m/s^2^). Activity was classified into 11 levels every 4 s (0.0, 0.5, and 1.0–9.0; 0.0 is less than 0.06 g). Activity level was converted to EE (kcal) using the following procedure. When the accelerometer detected three or more acceleration signals within 4 s, the activity was recognized as physical activity and classified as one of nine activity levels (1.0–9.0). Subsequently, EE was calculated as follows (Eq. 1) using Ka calculated from the weight (W) and the activity level^24^.

1 $$ {\text{EE}}\;\left( {{\text{kcal}}} \right) = {\text{Ka}} \times {\text{W}}\;\left( {{\text{kg}}} \right) $$

Ka is not disclosed because it is proprietary.

EE detected by the accelerometer was compared with measurements made with a respiratory chamber, and there was a significant correlation between walking EE (ρ = 0.928, p < 0.001) and non-walking EE (ρ = 0.477, p < 0.001), in agreement with an earlier study^24^.

### Research procedure

We calculated the sample size using G*Power. After obtaining consent from each patient who met the inclusion criteria, apathy scales were measured, and those with a score of ≥ 16 were excluded. The apathy scale comprises 14 items, and motivation is evaluated using a questionnaire in which each question is worth 0–3 points. A high score indicates low motivation, and a low score indicates high motivation. A patient with a score of ≥ 16 was considered as ‘demoralised’^23^. Data on the age, sex, height, weight, diagnosis, paralysed side, and Modified Rankin Scale (MRS) were obtained from electronic medical records. MRS describes six grades of disability after a stroke (grade 5 denotes severe disability, and grade 0 denotes no symptoms). We entered that the age, sex, height, and weight of each patient in the accelerometer. We ensured the patient wore the accelerometer on the designated part of the body.

For 3 days (starting from 9:00 am each day), the patient wore the accelerometer on the trousers of the non-paralysed limb or belt (upper anterior iliac spine), and EE (kcal) was recorded throughout the day except during bathing and sleeping. The purpose of the study was explained to the therapists in charge, and the intervention was implemented according to general guidelines.

Rehabilitation was conducted every day for 20–80 min per session. The presence of the accelerometer was confirmed, and the therapist recorded the start and end times of each session. The amount of physical activity was calculated by summing the EE measured every minute by the accelerometer from 9:00 am to 9:00 pm (bedtime) for each day and computing the average over 3 days. The participants were recruited in this study for 2 weeks. Data collection of the amount of physical activity of all participants was conducted from 9 November 2016 to 15 November 2016.

After measuring the activity for 3 days, each therapist evaluated physical function using FIM-M, BBS, and 10-m CWS.

### Physical functions

FIM, an evaluation scale for activities of daily living, rates functional independence. A total of 18 items, including 13 and 5 motor and cognitive items respectively, are evaluated on a 7-point scale. Seven, six, and five points indicates complete independence, no monitoring required, but auxiliary tools are required, and monitoring required, respectively; 4 points or less are given according to the amount of assistance. FIM-M has a total of 91 points corresponding to 13 items, including eight items on self-care, three items on transfer, and two items on movement.

BBS is a comprehensive evaluation of balance ability consisting of 14 items, including posture maintenance in sitting and standing positions, standing motion, one-leg standing, transfer motion, and direction change. Each item is scored on a scale of 0–4, for a possible total of 56 points.

The 10-m CWS was measured by setting a 10-m walkway and a 3-m front and back runway, for a total of 16 m. The therapist instructed the patient to walk at a comfortable speed. The measurement was started with a stopwatch from the moment the patient's sole crossed the start line and touched the ground and finished the moment the sole touched the ground beyond the end line.

### Statistical analysis

SPSS Statistics Ver23 (IBM Corp, Tokyo, Japan) was used for statistical analysis. The Shapiro–Wilk test was used to assess normality. Since the data were non-normally distributed, the Spearman’s rank correlation coefficient was used to test the relationship between groups. The significance level was set at α < 5%.

Since the scatter plots indicated that the EE range of patients with high function was wider than that of patients with low function, we stratified the patients into two groups for each index. For FIM-M, the group that was independent enough to not need monitoring in FIM gait items (6,7 points) was considered the high score group, and the group that required monitoring (5 points) was considered the low score group. Based on the criteria to determine the risk of falls^25,26^, patients with BBS score ≥ 46 points were classified as the high score group, and patients with ≤ 45 points were classified as the low score group. For walking speed, based on the criteria for indoor walking independence^27^, we considered the group faster than 0.27 m/s as the high score group and slower than 0.27 m/s as the low score group.

### Ethics approval and consent to participate

Study approval was obtained from the Shinshu University Medical Ethics Committee (approval number: 3555). All participants provided informed consent. All research was performed in accordance with relevant guidelines or regulations.

## Results

### Patient characteristics

Forty-six patients were selected; however, 4 patients did not give consent and were excluded. Of the remaining 42 patients, 6 were considered to have decreased motivation and were excluded. Of the 36 included patients, 25 were males and 11 were females; their average age was 66.2 ± 10.6 years. There were 1, 10, 4, and 21 participants with MRS scale 1, 2, 3, and 4, respectively (Table 1).

**Table 1:** Patient characteristics (n = 36).

Variable	Value
**Age (years)**	
Mean ± SD	66.2 ± 10.6
Median (range)	64 (46–86)
**Sex, n**	
Male	25
Female	11
**Period from onset (days)**	
Mean ± SD	55.2 ± 39.9
Median (range)	47.5 (15–211)
Cerebral haemorrhage, n (%)	13 (36)
Cerebral infarction, n (%)	23 (64)
**Paralysed side, n**	
Right	18
Left	18
**Modified Rankin Scale, n**	
1	1
2	10
3	4
4	21
**Body mass index, kg/m**^**2**^	
Mean ± SD	23.8 ± 4.5
Median (range)	22.8 (18.0–38.7)

### Relationship between physical function and EE

The median FIM-M, BBS, CWS, TEE, REE, and OEE were 75 points, 47 points, 0.62 m/s, 41.8 kcal, 18.5 kcal, and 16.6 kcal, respectively (Table 2). The correlations between FIM-M score, BBS score, CWS, TEE, REE, and OEE are shown in Table 3. There was a significant correlation between all physical functions and each EE type.

**Table 2:** Median and range of each physical function and energy expenditure (n = 36).

	Median	Range
FIM-M^a^ (point)	75	46–91
BBS^b^ (point)	47	24–56
CWS^c^ (m/s)	0.62	0.11–1.32
TEE^d^ (kcal)	41.8	2.9–152.3
REE^e^ (kcal)	18.5	0.1–114.1
OEE^f^ (kcal)	16.6	2.8–75.7

^a^Functional Independence Measure Motor score.

^b^Berg Balance Scale.

^c^10-m comfortable walking speed.

^d^Total energy expenditure per day.

^e^Energy expenditure during rehabilitation.

^f^Energy expenditure other than during rehabilitation.

**Table 3:** Correlation coefficient between physical function and energy expenditure.

	TEE^d^	REE^e^	OEE^f^
FIM-M^a^	0.424**	0.361*	0.436**
BBS^b^	0.592**	0.657**	0.465**
CWS^c^	0.544**	0.507**	0.499**

**p < 0.01, *p < 0.05.

^a^Functional Independence Measure Motor score.

^b^Berg Balance Scale.

^c^10-m comfortable walking speed.

^d^Total energy expenditure per day.

^e^Energy expenditure during rehabilitation.

^f^Energy expenditure other than during rehabilitation.

**Figure 1:**
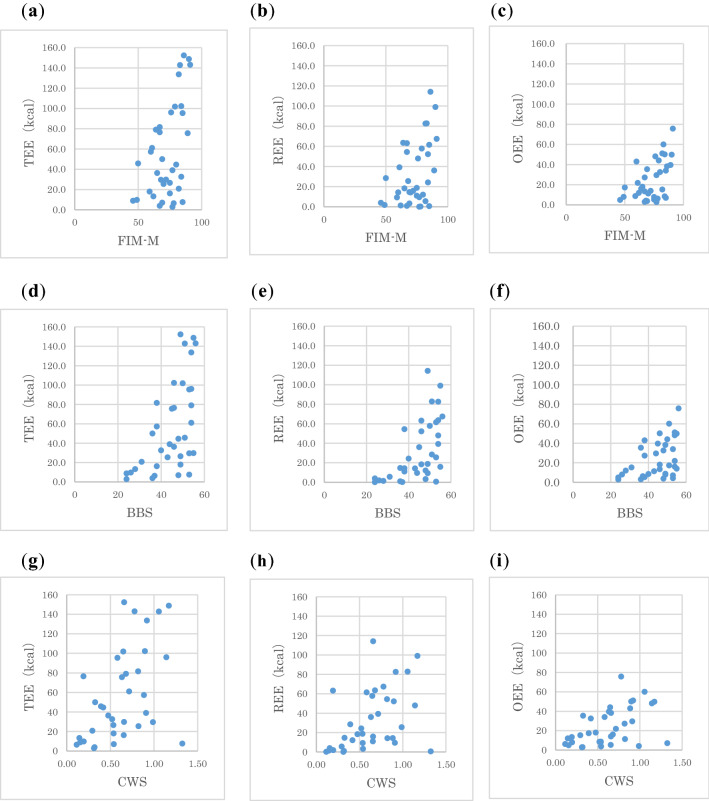
Scatter plots of physical function and energy expenditure (n = 36). Scatter plot of FIM-M and (**a**) TEE, (**b**) REE, and (**c**) OEE. Scatter plot of BBS and (d) TEE, (**e**) REE, and (**f**) OEE. Scatter plot of CWS and (**g**) TEE, (**h**) REE, and (**i**) OEE.

Figure 1 shows the scatter plot of physical function and TEE. Although there was a statistically significant correlation between physical function indices and EE, patients with high function had a wide range of EE compared to those with low function. It was visually confirmed that some of the patients were active, and some were barely active. This trend was observed between all EE and physical function indices.

### Relationship between physical function and EE stratified by physical function

The FIM-M high score group (TEE: ρ = 0.274, not significant [ns]; REE: ρ = 0.209, ns, OEE: ρ = 0.265, ns) and low score group (TEE: ρ = − 0.030, ns; REE: ρ = − 0.014, ns; OEE: ρ = − 0.045, ns) showed no significant correlation with any EE type (Table 4).

**Table 4:** Correlation coefficient between high and low levels of physical function and each energy expenditure (Spearman's rank correlation coefficient).

High low (n)	FIM-M^d^		BBS^e^		CWS^f^	
	High (15)	Low (21)	High (21)	Low (15)	High (31)	Low (5)
TEE^a^	0.274	− 0.030	0.291	0.691**	0.469**	0.500
REE^b^	0.209	− 0.014	0.302	0.675**	0.440*	0.700
OEE^c^	0.265	− 0.045	0.288	0.551**	0.436*	0.300

**p < 0.01, *p < 0.05.

^a^Total energy expenditure per day.

^b^Energy expenditure during rehabilitation.

^c^Energy expenditure except during rehabilitation.

^d^Functional Independence Measure Motor score.

^e^Berg Balance Scale.

^f^10-m comfortable walking speed.

In the BBS high score group, no significant correlation was observed (TEE: ρ = 0.291, ns; REE: ρ = 0.302, ns; OEE: ρ = 0.288, ns). whereas, in the BBS low score group, there was a significant correlation with all EE types (TEE: ρ = 0.691, p < 0.01; REE: ρ = 0.675, p < 0.01; OEE: ρ = 0.551, p < 0.01) (Table 4).

The CWS high score group showed a significant correlation with all EE types (TEE: ρ = − 0.469, p < 0.01; REE: ρ = − 0.440, p < 0.05; OEE: ρ = − 0.436, p < 0.05). However, no significant correlation was observed with any EE type in the low score group (TEE: ρ = − 0.500, ns; REE: ρ = − 0.700, ns; OEE: ρ = − 0.300, ns) (Table 4).

**Figure 2:**
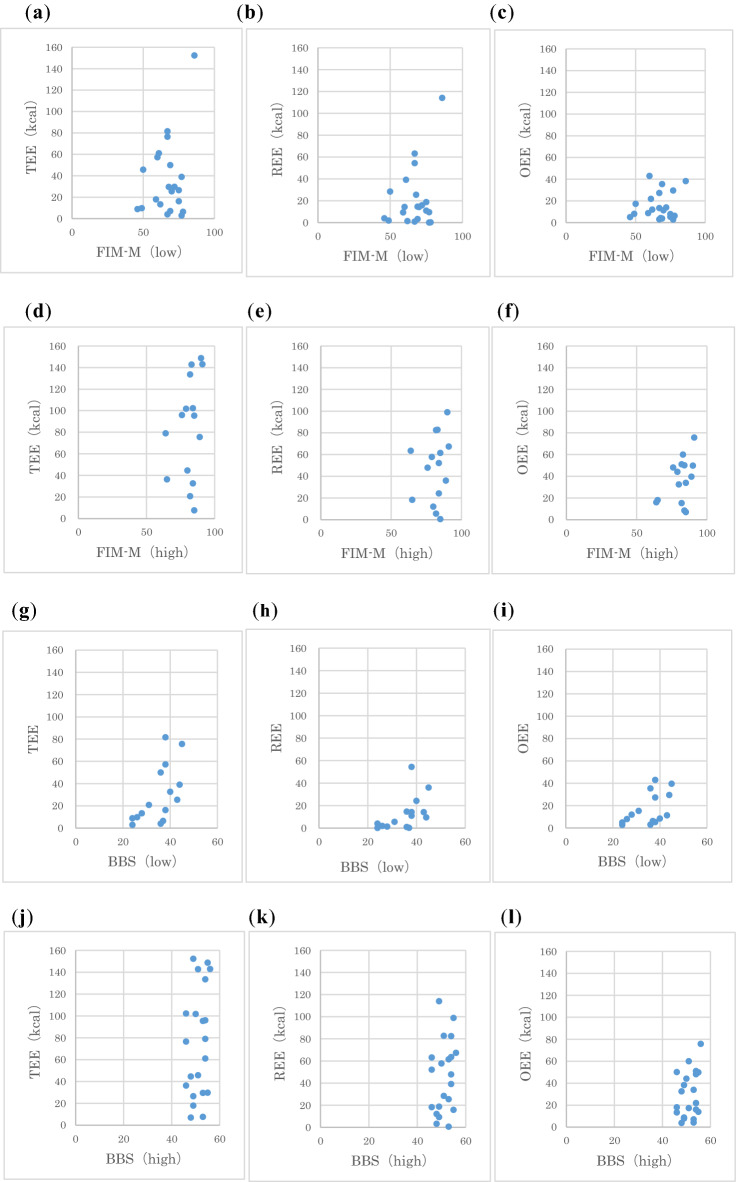
Scatter plots of physical function (high and low score groups of FIM-M and BBS) and energy expenditure. Scatter plot of FIM-M (low-score group, n = 21) and (**a**) TEE, (**b**) REE, and (**c**) OEE. Scatter plot of FIM-M (high score group, n = 15) and (**d**) TEE, (**e**) REE, and (**f**) OEE. Scatter plot of BBS (low score group, n = 15) and (**g**) TEE, (**h**) REE, and (**i**) OEE. Scatter plot of BBS (high score group, n = 21) and (**j**) TEE, (**k**) REE, and (**l**) OEE.

Figure 2 shows a scatter plot of both the FIM-M high score and low score groups as well as the BBS high score and low score groups. The CWS low score group had only five patients; therefore, the scatter plots for the high score and low score groups are not shown. No significant difference was noted in the scatter plot combining the high score and low CWS score groups.

## Discussion

Askim et al.^15^ previously reported that physical activity time in an upright position was positively correlated with improved physical function, including BBS and BI scores. Similar to rehabilitation, physical activities other than those performed during therapy affect physical ability. This study found a correlation between all EE types and physical function, supporting previous studies. Although we observed a correlation between EE and overall physical function, the trend differed based on ability level. A significant correlation was observed only in the low BBS group and in the high CWS group. However, no significant correlation was found between the other physical function subgroups and EE types.

The REE of several patients were similar to the OEE, irrespective of the time spent in rehabilitation. Additionally, there were several patients with an REE lower than the OEE. Therefore, it is highly possible that the therapy comprised mostly interventions in the lying or sitting position and few activities in the upright position. Conversely, the fact that some patients expended small REE despite the physical capacity to increase it suggests that upright position activity was not sufficiently administered. Therefore, physical function can be improved by increasing the upright position activity.

No significant correlation was noted with any form of EE in the FIM-M group, even after it was stratified into the high score and low score groups, suggesting that not enough energy commensurate with activities assessed with the FIM-M was expended throughout the day, even during rehabilitation. The high score FIM-M group consisted of patients who walk with self-support, a group with high physical function. Interestingly, there was a patient who walked independently whose REE was equivalent to the OEE of the FIM-M low score group, suggesting that the rehabilitation exercises were mostly in the lying or sitting position with few in the upright position. In addition, the OEE of most patients in the FIM-M high score group was equivalent to that of the FIM-M low score group, suggesting that there was little upright position activity during times other than rehabilitation. Therefore, it is recommended to provide lifestyle guidance on activities such as standing and walking to these patients.

In the low BBS group, which had a high risk of falling such that several patients needed monitoring during walking, there was a significant correlation with every EE index, suggesting that enough energy was being expended on balance. However, several patients in the low BBS group had an REE equivalent to the OEE of patients in the low FIM-M group. It is therefore highly likely that upright position activity was insufficient during rehabilitation. Moreover, most patients in the low BBS group had an OEE equivalent to that of the low FIM-M group, who also required monitoring during walking. Conversely, in the high BBS group, no correlation was found with any type of EE; thus, not enough energy was expended on balance. Although the high BBS group had good balance and a low risk of falling, there were patients in this group whose REE was equivalent to the OEE of patients in the low FIM-M group; hence, these patients may not be able to fully perform activities, such as walking during rehabilitation. The OEE of patients in the high BBS group was similar to that of patients in the low FIM-M group, even though their balance was good, suggesting that they did not spend much non-rehabilitation time performing activities in the upright position.

In the high CWS group, there was a significant correlation with every type of EE. Each EE index was expended throughout the day, during rehabilitation, and at times other than rehabilitation, commensurate with physical function. The number of patients in the low CWS group was small, and there was no correlation with any EE index.

In this study, some groups showed a correlation; however, it does not necessarily mean that an appropriate amount of EE was achieved. The amount of physical activity recommended for older adults is 1,000 kcal or more per week (142 kcal or more per day). The median TEE of our study, which is the EE per day, was 41.8 kcal. One of the reasons for the low REE is that a therapist cannot been able to develop rehabilitation content using the amount of physical activity as an index to increase the amount of activity^28^. High-intensity (60%–80% heart rate reserve) exercise improves physical function in stroke patients, and risk management can ensure low risk even at high intensity^29^. It is necessary to understand the complications and conditions of the patient and to intervene while considering the target heart rate into consideration. In addition, a previous study^30^ comparing video-recorded therapy time during actual rehabilitation with therapist-reported therapy time reported that therapists overestimated active therapy time and underestimated rest time. Therefore, the therapist must consider that he/she cannot provide a more active rehabilitation than he/she perceives. Therefore, it is useful to use a device that can perform an objective evaluation as used in this study.

To increase OEE, It is necessary to secure not only nurses but also caregivers, such as the patient's family, and develop a programme with the therapist using the amount of physical activity as an index. Further, it is necessary for the patients themselves to understand the importance of the amount of activity and consider programme planning that allows even one patient to increase the amount of activity without risk. Moreover, there is a report^31^ that a rich environment enhances activities, and it is necessary to improve the environment for enhancing activities in cooperation with facilities.

The results of this study were similar to those of previous studies, and there was an association between physical function and physical activity in stroke patients^9,10^; interestingly, the physical activity of the participants was inadequate^11–14^. In other words, in stroke patients, this tendency is similar and generalizability is considered to be present.

This study has some limitations. First, the single-axis accelerometer underestimates the actual EE by 8%^24^. Although the reliability of the single-axis accelerometer for gait in hemiplegic patients has been clarified, the severity of hemiplegic patients remains unknown. Second, this study has a small sample size, and the results are only from one facility; hence, other facilities should be included in future research. Finally, since this study is a cross-sectional study, it was impossible to examine the causal relationship; therefore, future studies should adopt a longitudinal design to evaluate the effect of EE in an upright position on physical function in subacute stroke patients.

In conclusion, we found that EE was correlated with overall physical function indices, however, the trend differed depending on the level of physical function. When patients were stratified based on this level, several groups showed no significant correlation. Therefore, several patients were unable to achieve EE in an upright position commensurate with their physical ability. However, even when a correlation is found between each physical function index and EE type, it does not necessarily mean that an appropriate amount of EE was achieved, as the EE of the patients in this study was probably less than recommended. Nevertheless, the median values of TEE (41.8 kcal), REE (18.5 kcal), and OEE (16.6 kcal) obtained from the accelerometer in this study can be used as references in future research.

## Supplementary Information

Supplementary Information.
