# Supplementary material for: The relationship between energy expenditure and physical functions in patients hospitalised for stroke
Source: Sci Rep. 2021 Nov 4;11:21685. doi: 10.1038/s41598-021-01135-3 (PMC8568978; doi:10.1038/s41598-021-01135-3)
Supplement: Supplementary file 1 — Supplementary Information. [file 41598_2021_1135_MOESM1_ESM.docx]

Walkable subacute stroke patients in hospital（n＝46）

Excluded from study（n＝4）：

・Subjects who did not agree with the research method（n＝4）

Subjects who gave consent

（n＝42）

Excluded from study（n＝6）：

・Apathy scale score of 16 points or more

（n＝6）

Activity measurement（n＝36）

Evaluation of each physical function

Statistical analysis

Supplementary Figure 1. Flow chart of patient selection
